# Epidemiological Characteristics of Intestinal Protozoal Infections and Their Risk Factors in Malaysia: Systematic Review and Meta-Analysis Protocol

**DOI:** 10.2196/66350

**Published:** 2025-04-04

**Authors:** Nor Shazlina Mizan, Hassanain Al-Talib, Seok Mui Wang

**Affiliations:** 1 Institute for Medical and Molecular Biotechnology Faculty of Medicine Universiti Teknologi MARA Sungai Buloh Malaysia; 2 Department of Medical Microbiology and Parasitology Faculty of Medicine Universiti Teknologi MARA Sungai Buloh Malaysia; 3 Cardiovascular Advancement and Research Excellence Institute (CARE Institute) Universiti Teknologi MARA Sungai Buloh Malaysia; 4 Non-Destructive Biomedical and Pharmaceutical Research Center Smart Manufacturing Research Institute Universiti Teknologi MARA Puncak Alam Malaysia

**Keywords:** intestinal protozoa, infection, gastroenteritis, epidemiology, parasite, risk factor, Malaysia, contamination, diarrhea, outbreak, socioeconomic, sanitation, systematic review, meta-analysis, protocol, observational, PRISMA

## Abstract

**Background:**

Intestinal protozoal infections caused by *Entamoeba histolytica, Giardia lamblia*, and *Cryptosporidium parvum* are prevalent in Malaysia. They cause severe diarrheal diseases with symptoms such as bloody stools, abdominal pain, stomach discomfort, and bloating. These infection outbreaks have been reported in diverse socioeconomic backgrounds and geographical regions usually during the rainy season or in areas with poor sanitation. Despite the importance of these infections, data on its overall prevalence, risk factors, and diagnostic methods remain limited.

**Objective:**

The aim of this study is to systematically review and synthesize evidence on the risk factors, prevalence, and detection methods for intestinal protozoal infections in Malaysia, offering insights that are applicable to other tropical and low-income regions.

**Methods:**

Studies on intestinal protozoal infections among Malaysian patients published after January 2010 up to November 2024 will be eligible for inclusion. The eligibility criteria include studies investigating infections caused by *E. histolytica, G. lamblia,* and *C. parvum* using validated diagnostic methods such as microscopy, molecular techniques, or immunoassays. Case reports, reviews, and studies without original data will be excluded. Comprehensive database searches will be conducted in PubMed/MEDLINE, Scopus, ProQuest, Web of Science, Google Scholar, and the Cochrane Library. The reference lists of selected papers are also checked. A standardized data extraction form will be used to record study characteristics, outcomes, and associated variables. Risk of bias will be assessed using the Joanna Briggs Institute tools and Newcastle-Ottawa Scale approach. Data synthesis will utilize a random effects model to estimate pooled prevalence and identify risk factors associated with these infections. Subgroup analyses will examine variations by geographic region and diagnostic method. Statistical heterogeneity will be assessed using *I*^2^ statistic and meta-regression. Publication bias will be assessed using Egger and Begg funnel plot test. The results are reported in accordance with PRISMA (Preferred Reporting Items for Systematic Reviews and Meta-Analyses) guidelines.

**Results:**

This systematic review was funded in June 2024. Database searches were started in July 2024, and we identified 1652 papers as of December 2024 for screening. Completion of study screening is anticipated by May 2025, with data extraction and analysis expected to conclude by December 2025.

**Conclusions:**

Our study will address critical knowledge gaps in the epidemiology and risk factors of intestinal protozoal infections in Malaysia. Study limitations include potential bias in study selection, heterogeneity in diagnostic methods, and differences in the reporting quality of the included studies. Our findings will provide valuable insights into the prevalence of these infections, the associated risk factors, and the diagnostic techniques employed, which should strengthen public health measures, improve diagnostic procedures, and guide future research to reduce the prevalence of intestinal protozoal infections in Malaysia.

**Trial Registration:**

PROSPERO (International Prospective Registry of Systematic Reviews) registration CRD42023456199; https://www.crd.york.ac.uk/PROSPERO/view/CRD42023456199

**International Registered Report Identifier (IRRID):**

DERR1-10.2196/66350

## Introduction

Intestinal protozoal infections are the most common parasitic infections and are a serious global health concern. An estimated 3.5 billion people are affected, with approximately 450 million individuals currently experiencing intestinal protozoal infections [1]. The typical intestinal protozoal infection cases are reported from *Entamoeba histolytica, Giardia lamblia,* and *Cryptosporidium parvum*. The transmission of these protozoa occurs via the oral-fecal route, which involves either indirect or direct contact with the infection. This includes various modes of transmission such as human-to-human, zoonotic, waterborne, and foodborne transmission [2,3]. The most common symptoms of an intestinal protozoal infection include nausea and watery diarrhea, which are caused by the release of enterotoxins, and are often accompanied by inflammation in the stomach, small intestine, and large intestine [3]. Individuals infected with *E. histolytica*, the causative agent of amoebiasis, show characteristic symptoms such as abdominal pain, bloody diarrhea, fever, and in severe cases, liver abscesses. *G. lamblia* causes giardiasis, which is characterized by watery diarrhea, abdominal pain, flatulence, and weight loss. Similarly, *C. parvum,* which is responsible for cryptosporidiosis, primarily manifests with watery diarrhea accompanied by stomach cramps, nausea, vomiting, and fever, with an increased risk in immunocompromised individuals. These different clinical signs emphasize the importance of targeted diagnostic approaches and tailored therapeutic interventions, with the severity of the disease depending on the immune status of the affected individuals [4,5]. The causes can be difficult to determine because the majority of enteric pathogens cause similar symptoms. There can be multiple infectious agents that can cause acute gastroenteritis, and contamination may come from food, water, the environment, or animals. Therefore, it is difficult to make accurate and fast reports via epidemiological analysis [6,7].

The primary method for detecting intestinal protozoa is the microscopic analysis of stool samples, which is laborious and requires specialized personnel. Molecular approaches such as real-time polymerase chain reaction offer increased sensitivity and efficiency and require only 1 sample for testing. Immunodiagnostic approaches targeting parasite antigens or host antibodies improve sensitivity and specificity. Despite advances, there is no one-size-fits-all strategy for diagnosis, which requires careful evaluation of aspects such as the suspected parasite, the nature of the sample, and the resources available [8]. A comprehensive approach may involve a combination of microscopic, molecular, and immunodiagnostic approaches to ensure accurate detection and treatment of intestinal protozoal infections.

Currently, intestinal protozoa, including *E. histolytica*, infect nearly 50 million individuals annually, leading to 100,000 deaths. Moreover, *Entamoeba* infection affects 50% of the global population, notably in Central and South America, Africa, and Asia, with rates up to 25% in certain limited-income and heavily indebted poor countries [9]. Giardiasis, caused by *G. lamblia*, is prevalent in the United States, the United Kingdom, Africa, Asia, and Latin America, and affects approximately 200 million people annually. In temperate countries such as Spain, the United Kingdom, and France, the prevalence in children is approximately 1.1%-2.1% [10,11]. *Cryptosporidium spp.* infection is widespread in the United States and tropical countries, with rates as high as 13% in India and 7.3% in Thailand [12,13]. These infections impose a significant burden, especially in heavily indebted poor tropical nations and parts of Central/South America, Africa, and Asia. Addressing sanitation and hygiene is crucial in prevention efforts.

In Malaysia, helminths and protozoa are the most common types of parasites causing diarrheal diseases. Diarrheal diseases are the leading cause of death in children younger than 5 years, with a mortality rate of 0.8% in 2019 [14]. The occurrence and wide distribution of intestinal protozoal infections in Malaysia represent significant events in the epidemiology of infectious diseases. Unfortunately, only limited data are currently available on the epidemiology and the risk factors associated with these diseases. Moreover, overall data on the diagnostic approach used to detect intestinal protozoal infections in Malaysia are lacking [15]. To address this knowledge gap, we will conduct a comprehensive systematic review with meta-analysis, aiming to synthesize the available data and overcome the limitations of individual studies. This study will be conducted to gain a better understanding of the prevalence and distribution of different intestinal protozoal infections, risk factors associated with intestinal protozoal infections among patients, and update on the diagnostic methods primarily used to detect intestinal protozoal infections, with a specific focus on the Malaysian population. Although this study focusses on Malaysia, the outcomes are also relevant for other tropical regions with similar socioeconomic, environmental, and health challenges. These findings can guide strategies to manage intestinal protozoal infections in regions such as Southeast Asia and beyond.

## Methods

### Patient and Public Involvement Statement

This protocol was not developed with the involvement of patients.

### Study Design

The systematic review and meta-analysis will be reported in accordance with PRISMA (Preferred Reporting Items for Systematic Reviews and Meta-Analyses) statement, which was considered in the development of this protocol (Multimedia Appendix 1) [16]. This protocol is registered with PROSPERO (International Prospective Registry of Systematic Reviews; registration CRD42023456199). The methodology chosen for this study is designed to be replicable in different settings. Although the focus is on Malaysia, the search strategy and data extraction can also be applied to other regions to gain comparable insights.

### Study Area

We will include studies published on intestinal protozoal infections among patients in Malaysia, except for case reports, reviews, and studies without original data, and the studies will be divided according to region. Malaysia is an ideal case study for the study of protozoal infections due to the unique intersection of tropical climate, diverse population, and varied socioeconomic conditions. These factors combined with the limited epidemiological data underscore the urgent need for comprehensive research in this context.

### Types of Participants

We will include primary studies that investigate patients without age restrictions that provide estimates of any symptoms of intestinal protozoal infection (eg, amoebiasis, giardiasis, cryptosporidiosis). We will include studies that explicitly describe the detection methods and definitions of intestinal protozoal parasites. Studies on intestinal protozoal infections in which at least one of the 3 protozoa were detected will be included. We will exclude case reports, reviews, and studies without original data. If we find multiple studies with the same data, then we will use the study with the largest sample size. The results will be presented in tables and figures.

### Types of Outcome Measures

This meta-analysis will have 2 main outcomes. The primary outcome will be the prevalence of intestinal protozoal parasites in Malaysia. The second aim is to investigate the risk factors associated with the diseases and the update on the primarily used detection methods for intestinal protozoa, that is, *C. parvum, E. histolytica,* and *G. lamblia*.

### Information Sources

The following electronic databases of scientific literature that have been peer-reviewed, as recommended by Siddaway et al [17], will be carefully searched without language restrictions and limited to human research only: PubMed/MEDLINE, Scopus, ProQuest, Web of Science, Science Direct, Google Scholar, and Cochrane Library databases. A manual search of reference lists of identified papers will be added to the electronic searches. The authors of the research will be contacted for explanation or to provide any additional crucial missing metadata when necessary. Google Scholar will be used meticulously to search for relevant literature, including grey literature and unpublished data, to compile a thorough understanding of the prevalence rates, socioeconomic impact, and various diagnostic techniques employed for these infections within the Malaysian population. Studies completed after January 1, 2010, with no language constraints will be considered.

### Search Strategy

The proposed search term for the first theme will be developed in MEDLINE by using medical subject headings (MeSH) combined with free-text terms around the 3 search components for intestinal protozoal infection, that is, giardiasis, cryptosporidiosis, and amoebiasis. The second theme will be prevalence, including epidemiology, incidence, epidemiological studies, and observational studies. The third theme will be risk factors and detection methods of intestinal protozoal infection and other similar names used (see detailed list in Multimedia Appendix 2) and then adapted for use in the other databases. The search, the evaluation of the titles and abstracts, and the review of the complete texts will be performed independently by an author. After removing duplicates and irrelevant entries, the reference lists of the studies received were checked for further studies that were not found in the database search.

### Eligibility Criteria

The selection or exclusion of studies is based on the criteria proposed in Table 1. Studies published after January 2010 up to November 2024 will be eligible for inclusion. The literature search is limited to papers published after 2010, highlighting the significant change in the diagnostic process when molecular techniques began to replace traditional microscopic methods for detecting intestinal protozoal infections. This time frame allows for studies that include both the transition period and the continuous use of microscopy, including the more recent implementation of molecular techniques. This methodology allows for a full assessment of the evolution of diagnostic methods across time. We will not apply any language restrictions as part of the eligibility criteria. The PICO (Population, Intervention, Comparison, Outcome) classification technique is a popular method for creating search methods and describing meta-analyses.

The study population includes individuals with intestinal protozoal infections identified through positive laboratory test results, physician diagnoses, self-reported infection status, treatment registries, or other medical records. Both asymptomatic and symptomatic cases will be included, with no restrictions on age, gender, ethnicity, or geographical location within Malaysia. Eligible populations encompass mixed communities, outpatients, inpatients, and residential settings, spanning diverse socioeconomic backgrounds. This inclusion criterion ensures a representative sample of intestinal protozoal infections across Malaysia.

**Table 1:** Criteria for inclusion and exclusion.

Category	Included	Excluded
Population	Both asymptomatic and symptomatic cases from diverse socioeconomic backgrounds, including all ages, genders, ethnicities, and geographical locations in Malaysia (including non-Malaysians) will be considered, with identification based on laboratory tests, physician diagnoses, or medical records	Both asymptomatic and symptomatic cases conducted outside Malaysia or non-Malaysian settings will be excluded, including cases from mixed communities, outpatient, inpatient, and residential settings, regardless of age, gender, ethnicity, or non-Malaysians
Intervention/exposure	Studies conducted on the 3 common intestinal protozoal parasites (*Entamoeba histolytica, Giardia lamblia*, and *Cryptosporidium parvum*) and using any of these methods: microscopic examination, immunoassay technique, and molecular methods	Studies excluding these 3 intestinal protozoa (*E. histolytica, G. lamblia*, and *C. parvum*) and other intestinal protozoa parasites unrelated to this study or using other detection methods as mentioned
Study design	Quantitative studies, cross-sectional studies, cohort, and case controls will be included	Case reports, reviews, and studies without original data will be excluded
Outcome	Diarrhea and presence of intestinal protozoa	Nonenteric infection and studies without detection method mentioned

The primary outcome measure is self-reported diarrhea, which is commonly defined as the occurrence of 3 or more watery stools or bloody diarrhea within a 24-hour period. This information is reported by the patient’s family or caretaker and covers a 2-week period preceding the assessment. Additionally, laboratory confirmation of diarrheal illness involves the identification of specific pathogens in the patient’s stool. This confirmation can be achieved through microscopic examination or culture techniques. Furthermore, the presence of intestinal parasites is confirmed through laboratory procedures, including microscopy, serological tests, or molecular methods.

This study will focus on the prevalence of intestinal protozoal infections in Malaysia, exploring the epidemiology, diagnostic methods, and contributing factors to these infections. This research aims to investigate the extent to which individuals in Malaysia are affected by these parasitic infections and the various factors contributing to their occurrence, shedding light on their epidemiology and diagnostic methods. We will consider both randomized and nonrandomized controlled trials, along with observational studies that have examined the connection between the exposure and outcome of interest. We will collect data on the measures of association. However, we will put a lower priority on unadjusted measures (please refer to the “Risk of bias assessment for eligible studies” section for details).

### Screening and Selection Procedure

**Figure 1:**
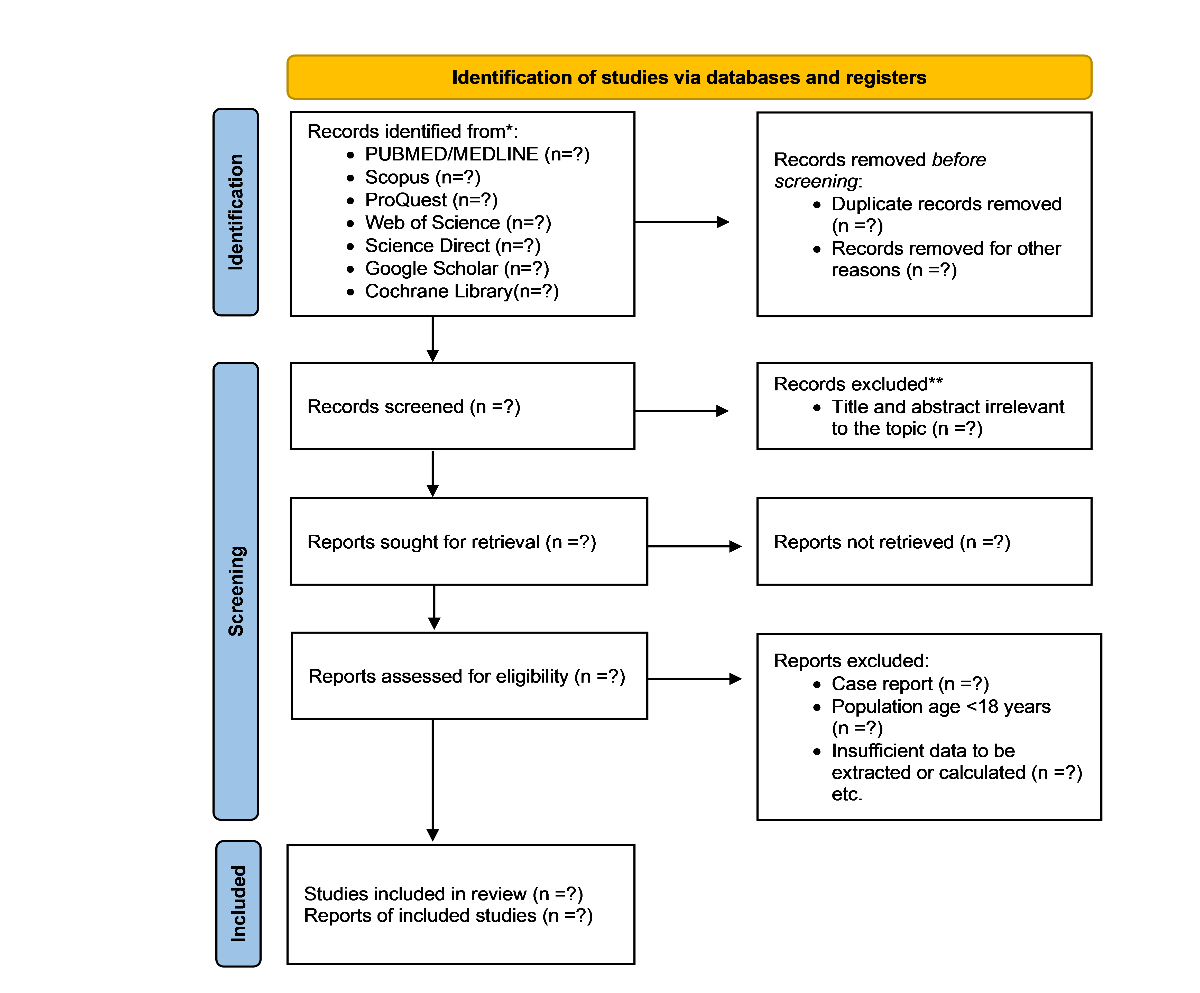
PRISMA (Preferred Reporting Items for Systematic Reviews and Meta-Analyses) flow diagram for systematic review and meta-analysis. *Reporting the number of records identified from each database or register searched (rather than the total number across all databases/registers). **Indicate how many records were excluded by a human and how many were excluded by automation tools.

In the research methodology, citations retrieved from diverse search engines will be imported into EndNote and subjected to a deduplication process to eliminate redundant entries. The exclusion criteria are usually unrelated, duplicate, unavailable full texts, or pure abstracts. These exclusions should be stated in advance to prevent any biases. The inclusion criteria would be studies with the target patients, interventions studied, or the comparison between the 2 interventions studied. These are publications that contain information relevant to our study subject. The most critical aspect is that the information provided is clear and adequate to answer the problem, whether positive or negative. Then, the required data will be meticulously extracted by the primary researcher (NSM). To ensure accuracy and reliability, the extracted information from all papers will undergo a thorough cross-checking process conducted independently by 2 other researchers (HA-T and SMW). The goal of this review is to confirm the consistency of the data, identify and mitigate potential biases, and correct minimal errors. The validated studies will be organized and stored in a structured spreadsheet program specifically designed for systematic data extraction. Following this, the collected data will undergo a series of rigorous assessments to evaluate the adequacy of the studies, ensuring a solid and reliable basis for subsequent analysis and interpretation. We will record the selection process in sufficient detail to complete a PRISMA flow diagram shown in Figure 1 [18,19].

### Data Extraction

We will use a standardized data extraction created in the online Microsoft Excel spreadsheet for study characteristics and outcome data. The data capture sheet will consist of a screening checklist consisting of study details (author, year of publication, period of study, place the study was performed), study characteristics (sample size, mean age, and age range of participants), risk factor analysis (socioeconomic status, population characteristics, geographical area), and outcome (presence of intestinal protozoa status; odds ratios, prevalence ratios, relative risks or hazard ratios [95% CI] with their related variability [SD or SE]). Please see “Eligibility criteria” section and Table 1 for the list and definitions of the aforementioned exposures and outcomes. Any significant limitations of the study will be identified, and any further relevant information will be requested from the corresponding author if necessary.

### Data Synthesis

The data will be analyzed using STATA software (version 17.0; William Gould) and REVMAN software (The Cochrane Collaboration). To accurately report the content of each paper under consideration and to explore the relationships between outcomes and risk factors, the data will be summarized in a tabular form and the main findings of each paper will be presented, including descriptive analysis of gender, age, region, and others. We will also calculate the overall prevalence of intestinal protozoal infections among patients by using a random effects model to allow heterogeneity across the included studies and later divide the studies into regions. This study will include a qualitative or narrative analysis to summarize the key features and overall quality of the publications. In addition, a comprehensive assessment of potential bias will be performed for each paper (see the “Assessment of Risk of Bias for Eligible Studies” section). All meta-analyses will be conducted using the random effects model because of the expected epidemiologic and clinical heterogeneity. Because the underlying effects of the different studies are assumed to be normal, we will use random effects modelling to account for variation within and between studies.

### Risk of Bias Assessment for Eligible Studies

Risk of bias (quality assessment) will be performed using the Joanna Briggs Institute critical appraisal tools [20]. The primary author and 2 independent reviewers will evaluate each study individually at both the outcome and study levels to provide an overall assessment of the risk of bias. Each reviewer will examine each study as yes, no, unclear, or not applicable for bias (Multimedia Appendix 3). The quality of the included studies will be assessed using the Newcastle-Ottawa Scale, which is specifically designed to evaluate observational and cohort studies [21]. This tool will ensure a consistent and balanced evaluation of the methodological quality of all studies included in the review, accommodating the variability between experimental and observational study designs (Multimedia Appendix 4).

### Assessment of Heterogeneity

We will visually inspect the forest plots for any evidence of heterogeneity. The pooled estimates will be visually assessed using forest plots to determine the degree of heterogeneity between studies. We will also measure statistical heterogeneity by using the *I*^2^ statistic to estimate the difference between studies [22]. The *I*^2^ statistic quantifies the proportion of variability observed in the prevalence estimates that can be explained as genuine variations in treatment effects rather than random sampling error. The *I*^2^ statistic is bounded in the scientific literature by a range of 0% to 100%. Values between 0% and 25% indicate low heterogeneity, while values between 75% and 100% indicate substantial heterogeneity [23]. We will investigate potential sources of heterogeneity by using subgroup analyses and meta-regression. The possible causes will be explored and evaluated in terms of their methodological characteristics to determine whether the degree of heterogeneity can be explained by differences in these characteristics and whether meta-analysis is appropriate. The overall prevalence and 95% CIs estimate of the outcome (presence of intestinal protozoa status; odds ratios, prevalence ratios, relative risks or hazard ratios), with their related variability (SD or SE) will be presented in forest plots. If the selected studies are acceptable for quantitative synthesis, the data will be pooled in a meta-analysis to combine the primary exposures across studies and to provide a summary of effects according to study design/measurement of effects. Where possible, we will also conduct subgroup analyses by age group, detection methods (microscopy, immunoassays, and molecular techniques), study design (ie, cross-sectional or prevalence studies), and geographical region (Peninsular Malaysia and East Malaysia) to assess differences between the strength of association by geographical location and the potential impact of contextual confounders, which may vary by region.

### Meta-Biases

To assess the possibility of publication bias, we plan to use funnel plots [24], a widely used graphical tool in meta-analyses, to visually check the symmetry of the data distribution. In addition, we will apply statistical tests such as Egger and Begg funnel plot test [25], which are specifically designed to detect asymmetries in funnel plots and provide quantitative measures of publication bias. In addition, we intend to perform a comparative analysis between the outcomes derived from the fixed effects model and the random effects model. This comparative evaluation will allow us to assess the possible presence of small sample bias in the published literature. Specifically, we aim to determine whether the effects of the studied exposure appear to be more pronounced in studies with smaller sample sizes, indicating a possible bias in favor of smaller studies in the existing literature. By applying these comprehensive analyses, we aim to ensure the robustness and reliability of our results and to rule out potential biases that could affect the interpretation of the results.

### Ethical Considerations

No primary data will be collected; thus, no formal ethical approval is required. The results will be disseminated though a peer-reviewed publication and conference presentation.

## Results

This systematic review was funded in June 2024. Database searches were started in July 2024 using specific databases such as PubMed, Scopus, ProQuest, Web of Science, Science Direct, Google Scholar, and Cochrane Library databases. As of December 2024, 1652 studies have been identified for title and abstract screening. The screening phase is expected to be completed by May 2025. Data extraction and synthesis will be performed. Data analysis is expected to be completed by December 2025. The results will be published in peer-reviewed journals and presented at conferences in early 2026.

## Discussion

Currently, there are few epidemiological data on the prevalence of intestinal protozoal infections in the Malaysian population. A comprehensive study of the prevalence and distribution of intestinal protozoal infections is critical to understanding their public health impact and implementing effective control measures. Given the lack of available data, it is important that Malaysia conduct detailed epidemiological studies that will allow a more thorough assessment of the prevalence, risk factors, and distribution of these intestinal protozoa. Such studies would not only make an important contribution to the scientific knowledge base but also facilitate the development of targeted interventions and public health strategies to reduce the burden of these infections in the region. The results of this study will show that local data are consistent with global data, indicating that socioeconomic factors and diagnostic limitations are key barriers to the treatment of intestinal protozoal infections. For example, the diagnostic challenges in Malaysia mirror those observed in other tropical regions such as sub-Saharan Africa and South Asia. By addressing these gaps, this study provides a framework that can be transferred to other regions with similar public health problems.

The theory under study is that several factors, particularly in vulnerable populations such as low-income societies with poor sanitation and immunocompromised patients, may increase the risk of infection with intestinal protozoa and that differences in the validity of different diagnostic techniques may lead to different rates of detection of intestinal protozoa, that is, false positive or false negative results. The above hypothesis proposes that the combination of various contextual risk factors may jointly contribute to an increase in the overall prevalence of intestinal protozoal infections in the studied population.

In our study, we will conduct a thorough and systematic review, including a meta-analysis, to integrate the available data and address the limitations of individual studies. Our main objective is to better understand the prevalence and geographic distribution of intestinal protozoal disease. In addition, we will investigate the factor analysis that contributes to this infection and its relationship with its detection method. Our work, by synthesizing this extensive information, will provide a more nuanced and comprehensive view of intestinal protozoal infections and add significantly to the current body of knowledge in the field of infectious diseases and public health. No changes to the current protocol are envisioned. If critical changes prove necessary, they will be documented in the published review. Our findings will be published in a peer-reviewed scientific journal. In addition, they will be disseminated to relevant health care organizations.

A limitation of this study is that data on symptoms of intestinal protozoal infections are currently extracted from medical records, which may lead to underreporting due to gaps in documentation. Although documented medical records are often used as the main source of data, this approach has limitations. This method is consistent with real-life situations where diagnostic assessment often depends on accessible clinical data. Implementing a model that requires additional prospective assessments of patients beyond conventional clinical care procedures may be impractical, particularly in a health care setting where such assessments are often not mandatory. It is crucial to understand that the proposed model is preliminary and should not be used in clinical practice without additional validation. Another striking feature of our research design is the possibility of selection bias due to differences in duration of symptoms or time since exposure to protozoal infections at the time of study entry. By excluding individuals with recent protozoal infections who may have different clinical characteristics or outcomes, we aim to create a model that applies only to those who may have overcome the acute phase of infection.

In addition, future research should investigate the serial examination of clinical symptoms and use dynamic modelling techniques to enable the application of the model across different stages of infection. A comparison of the performance of the model we developed with established diagnostic algorithms or clinical guidelines in similar patient populations would also be crucial for validation. However, it should be noted that certain diagnostic indicators required for established algorithms may not be routinely assessed during standard clinical care, necessitating additional assessments beyond the scope of our study.

Even after further validation, predictive models for intestinal protozoal infections are primarily ambiguous, and the goal is to provide guidance to health care providers regarding potential infection outcomes and not to replace clinical assessment. Once the models are externally validated, a clinical decision aid should be available to better identify individuals at high risk for protozoal infections, thereby improving patient care and regulating appropriate diagnostic techniques. The future development of such a decision aid would require collaboration with health care professionals and researchers to ensure its effectiveness and appropriateness in clinical practice.

## Appendix

Multimedia Appendix 1 PRISMA-P (Preferred Reporting Items for Systematic Review Protocols) 2015 checklist.

Multimedia Appendix 2 MEDLINE search strategy.

Multimedia Appendix 3 Joanna Briggs Institute’s critical appraisal checklist.

Multimedia Appendix 4 Newcastle-Ottawa quality assessment scale.

## Abbreviations

MeSH: medical subject headings
PICO: Population, Intervention, Comparison, Outcome
PRISMA: Preferred Reporting Items for Systematic Reviews and Meta-Analyses
PROSPERO: International Prospective Registry of Systematic Reviews
