# Supplementary material for: Epidemiological Characteristics of Intestinal Protozoal Infections and Their Risk Factors in Malaysia: Systematic Review and Meta-Analysis Protocol
Source: JMIR Res Protoc. 2025 Apr 4;14:e66350. doi: 10.2196/66350 (PMC12008696; doi:10.2196/66350)
Supplement: Multimedia Appendix 1 [file resprot_v14i1e66350_app1.docx]

**APPENDIX 1**

**PRISMA-P (Preferred Reporting Items for Systematic review and Meta-Analysis Protocols) 2015 checklist: recommended items to address in a systematic review protocol [1].**

| Section and topic | Item No | Checklist item |
| --- | --- | --- |
| ADMINISTRATIVE INFORMATION | | |
| Title: |  |  |
| Identification | 1a | Epidemiological Characteristics of Intestinal Protozoan Infections and Their Risk Factors in Malaysia: A Systematic Review and Meta-Analysis Protocol |
| Update | 1b | Updating (with methodological modification) by (Wells et al., 2000; Wells et al., 2012) |
| Registration | 2 | PROSPERO registration number: CRD42023456199 |
| Authors: |  | Nor Shazlina Mizan^1^, Seok Mui Wang^2,3,4^ and Hassanain Al-Talib^2^*  ^1^Institute for Medical and Molecular Biotechnology (IMMB), Faculty of Medicine, Universiti Teknologi MARA (UiTM), Sungai Buloh, 47000, Selangor, Malaysia.;  ^2^Department of Medical Microbiology and Parasitology, Faculty of Medicine, Universiti Teknologi MARA (UiTM), Sungai Buloh, 47000, Selangor, Malaysia.;  ^3^Institute of Pathology, Laboratory and Forensic Medicine (I-PPerForM), Universiti Teknologi MARA (UiTM), Sungai Buloh, 47000, Selangor, Malaysia.  ^4^Non-Destructive Biomedical and Pharmaceutical Research Center, Smart Manufacturing Research Institute (SMRI), Universiti Teknologi MARA (UiTM), Puncak Alam, 42300, Selangor, Malaysia.  *****Correspondence: hassanain@uitm.edu.my; Tel.: (+060361267438) |
| Contact | 3a |  |
| Contributions | 3b | Conceptualisation, S.M. and H.A.; methodology, S.M. and H.A.; validation, S.M., H.A. and W.S.; formal analysis, H.A. and W.S.; investigation, S.M.; resources, S.M.; data curation, S.M.; writing the original draft, S.M. and H.A.; reviewing and editing; S.M.; visualisation, S.M.; monitoring, H.A. and W.S. The published version of the manuscript has been read and approved by all the authors. |
| Amendments | 4 | Significant protocol amendments will be updated in PROSPERO and reported in the systematic review and meta-analysis final paper. |
| Support: |  |  |
| Sources | 5a | 600-RMC/PRGS 5/3 (004/2024) |
| Sponsor | 5b | This study received fund from Universiti Teknologi MARA (UiTM) under Prototype Development Research Grant (PRGS) grant. |
| Role of sponsor or funder | 5c | None declared. [p*age 25]* |
| INTRODUCTION | | |
| Rationale | 6 | Intestinal protozoan infections including infections caused by *Entamoeba histolytica, Giardia lamblia*, and *Cryptosporidium parvum,* are prevalent among diarrheal patients in Malaysia. These three common intestinal protozoa cause severe diarrheal disease with symptoms such as bloody stools, abdominal pain, stomach discomfort and bloating. Outbreaks of these infections have been reported in diverse socioeconomic backgrounds and geographical regions, usually during the rainy season or in areas with poor sanitation. Despite its importance, data on overall prevalence, risk factors and diagnostic methods in Malaysia remain limited. *[page 7]* |
| Objectives | 7 | The aim of this study is to systematically review and synthesize evidence on the risk factors, prevalence and detection methods for these infections in Malaysia, offering insights that are applicable to other tropical and developing regions.  *[page 7]* |
| METHODS | | |
| Eligibility criteria | 8 | **Design article**: Randomized and non-randomized controlled trials, cross-sectional studies, cohort studies, and other observational studies investigating the presence of intestinal protozoan infections and their associated risk factors in Malaysia; Studies on intestinal protozoan infections among Malaysian patients published after January 2010 up to November 2024 will be eligible for inclusion. Studies reporting unadjusted measures of association will be included but given lower priority. Case series, case reports, duplicate publications, and studies not focused on the Malaysian population will be excluded.  **Population**: Representative sample of intestinal protozoan infections in population. Both asymptomatic and symptomatic. No restrictions on age, gender, ethnicity, or geographical location in Malaysia. Eligible populations include mixed communities, outpatient, inpatient, and residential settings, with diverse socioeconomic backgrounds. Intestinal protozoan infection patient identification by positive laboratory test, physician diagnosis, self-reported infection status, infection treatment registries, other medical/administrative registers.  **Outcome**: Prevalence of intestinal protozoan infections as confirmed through laboratory techniques (microscopy, culture, serological, or molecular methods). Primary outcomes include self-reported diarrhea, defined as three or more watery or bloody stools within 24 hours over a two-week period, and laboratory-confirmed identification of intestinal protozoa in stool samples. Studies that do not report prevalence or outcomes related to intestinal protozoan infections will be excluded.  *[page 11-15]* |
| Information sources | 9 | Searching in the electronic databases (PubMed/MEDLINE, Scopus, ProQuest, Web of Science (WOS), Science Direct, and Google Scholar, and Cochrane Library databases), manual references’ listing of included studies and relevant reviews and consultation with experts in the field. *[page 10]* |
| Search strategy | 10 | See Appendix 2 |
| Study records: |  |  |
| Data management | 11a | Studies retrieved from diverse search engines will be imported into EndNote and subjected to a deduplication process to eliminate redundant entries. A two-step independent process will be performed by main author assessing identified studies for eligibility. To ensure accuracy and reliability, the extracted information from all articles will undergo a thorough cross-checking process conducted independently by two other authors. The study selection process will be reported in a PRISMA flow diagram. *[page 15-16]* |
| Selection process | 11b | One independent reviewer up to the study selection phase of the review. Two supervised authors from the data extraction phase. *[page 16]* |
| Data collection process | 11c | Data extraction will be performed by one supervised author using a standardized spreadsheet. Up to three attempts by mail will be done if additional data or clarification will be required from the included studies. *[page 16]* |
| Data items | 12 | The data capture sheet will consist of a screening checklist consisting of study details (author, year of publication, period of study, place the study was carried out), study characteristics (sample size, mean age, and age range of participants), risk factor analysis (socioeconomic status, population characteristics, geographical area) and outcome (presence of intestinal protozoa status; odds ratios, prevalence ratios, relative risks or hazard ratios), with their related variability (standard deviations or errors [SD, SE respectively), and 95% confidence intervals [95% CI]). *[page 16-17]* |
| Outcomes and prioritization | 13 | 1. Overall prevalence data of intestinal protozoan parasites in Malaysia.; ii) Overall prevalence data of each intestinal protozoan parasites in Malaysia.; iii) Overall prevalence data based on subgroup; iv) risk factors associated with the diseases; v) update on the primarily used detection methods for intestinal protozoa. *C. parvum, E. histolytica* and *G. lamblia*. *[page 9-10]* |
| Risk of bias in individual studies | 14 | Two different instruments will be applied: i) the Joanna Briggs Institute’s critical appraisal checklist, [4] and ii) Newcastle-Ottawa Scale approach [2,3]. The Joanna Briggs Institute’s checklist will be used as a reference when conducting sensitivity analysis restricted to high quality studies while the Newcastle-Ottawa Scale approach will be assed for the agreement. *[page18]* |
| Data synthesis | 15a | We will estimate the overall prevalence of intestinal protozoan infection among patients in specific settings according to the different population characteristic based upon the sampling method used and the relevant context using a random effect model to allow heterogeneity across the included studies and later divide the studies into regions. The study will include a qualitative or narrative analysis to summarise the key features and overall quality of the publications. *[page 17-18]* |
|  | 15b | Intestinal protozoan infection prevalence pooled estimates for all *pre-specified* outcomes will be computed applying random effect meta-analysis models. Heterogeneity within included studies will be assessed using the I^2^ statistic and visual inspection of forest plots. *[page 18-19]* |
|  | 15c | We will investigate potential sources of heterogeneity using subgroup analyses and meta-regression. The possible causes will be explored and evaluated in terms of their methodological characteristics to determine whether the degree of heterogeneity can be explained by differences in these characteristics and whether meta-analysis is appropriate. [page 19] |
|  | 15d | Descriptive analysis and report the characteristics of included studies. *[page 19]* |
| Meta-bias(es) | 16 | To assess publication bias, we will use funnel plots to visually examine data symmetry and apply Egger's and/or Begg's tests to detect asymmetries. Additionally, we will compare outcomes from the fixed effects and random effects models to evaluate small sample bias, particularly whether smaller studies show exaggerated effects, indicating potential bias. These analyses will help ensure the robustness and reliability of our results, minimizing the impact of biases on interpretation. *[page 20]* |
| Confidence in cumulative evidence | 17 | NA |

*** It is strongly recommended that this checklist be read in conjunction with the PRISMA-P Explanation and Elaboration (cite when available) for important clarification on the items. Amendments to a review protocol should be tracked and dated. The copyright for PRISMA-P (including checklist) is held by the PRISMA-P Group and is distributed under a Creative Commons Attribution Licence 4.0.**

*From: Shamseer L, Moher D, Clarke M, Ghersi D, Liberati A, Petticrew M, Shekelle P, Stewart L, PRISMA-P Group. Preferred reporting items for systematic review and meta-analysis protocols (PRISMA-P) 2015: elaboration and explanation. BMJ. 2015 Jan 2;349(jan02 1):g7647.*

**REFERENCES**

1. Moher D, Shamseer L, Clarke M, Ghersi D, Liberati A, Petticrew M, Shekelle P, Stewart LA, Prisma-P Group. Preferred reporting items for systematic review and meta-analysis protocols (PRISMA-P) 2015 statement. Systematic reviews. 2015 Dec;4:1-9.
