# Supplementary material for: Epidemiological Characteristics of Intestinal Protozoal Infections and Their Risk Factors in Malaysia: Systematic Review and Meta-Analysis Protocol
Source: JMIR Res Protoc. 2025 Apr 4;14:e66350. doi: 10.2196/66350 (PMC12008696; doi:10.2196/66350)
Supplement: Multimedia Appendix 2 [file resprot_v14i1e66350_app2.docx]

**APPENDIX 2**

*Medline search strategy*

- Intestinal Protozoan Infection (Population):

1. Intestinal Protozoan Infections [MeSH]
2. Giardiasis [MeSH]
3. Cryptosporidiosis [MeSH]
4. Amebiasis [MeSH]
5. Giardia* [tiab]
6. *Cryptosporidium**[tiab]
7. *Entamoeba** [tiab]

**Preferred search term;** “Intestinal Protozoan Infections"[MeSH] OR "Giardiasis"[MeSH] OR "Cryptosporidiosis"[MeSH] OR "Amebiasis"[MeSH]) AND ("Giardia*"[tiab] OR "Cryptosporidium*"[tiab] OR "Entamoeba*"[tiab])

- Epidemiology (Condition):

1. Epidemiology [MeSH]
2. Prevalence [MeSH]
3. Incidence [MeSH]
4. Epidemiological Studies [MeSH]
5. Cross-Sectional Studies [MeSH]
6. Observational Studies [MeSH]
7. Longitudinal Studies [MeSH]
8. Disease Outbreaks [MeSH]
9. Disease Surveillance [MeSH]
10. Risk Factor [MeSH]
11. prevalen* [tiab]
12. Inciden*[tiab]
13. Epidemiol* [tiab]
14. frequenc*[tiab]
15. occurrenc*[tiab]
16. morbidit*[tiab]
17. rate* [tiab]

**Preferred search term:** (("Epidemiology"[MeSH] OR "Prevalence"[MeSH] OR "Incidence"[MeSH] OR "Epidemiological Studies"[MeSH] OR "Cross-Sectional Studies"[MeSH] OR "Observational Studies"[MeSH] OR "Longitudinal Studies"[MeSH] OR "Disease Outbreaks"[MeSH] OR "Disease Surveillance"[MeSH] OR "Risk Factor"[MeSH]) AND ("prevalen*"[tiab] OR "Inciden*"[tiab] OR "Epidemiol*"[tiab] OR "frequenc*"[tiab] OR "occurrenc*"[tiab] OR "morbidit*"[tiab] OR "rate*"[tiab]))

- Detection Methods:

1. Diagnostic Techniques Digestive System [MeSH]
2. Microscopy [MeSH]
3. Molecular Diagnostic Techniques [MeSH]
4. Polymerase Chain Reaction MeSH]
5. Immunologic Tests [MeSH]
6. Stool Examination [MeSH]
7. Serologic Tests [MeSH]
8. Microscopy [tiab]
9. Polymerase Chain Reaction [tiab]
10. Immunologic Tests [tiab]
11. Stool Examination [tiab]
12. Serologic Tests [tiab]

**Preferred search term:** (("Diagnostic Techniques Digestive System"[MeSH] OR "Microscopy"[MeSH] OR "Molecular Diagnostic Techniques"[MeSH] OR "Polymerase Chain Reaction"[MeSH] OR "Immunologic Tests"[MeSH] OR "Stool Examination"[MeSH] OR "Serologic Tests"[MeSH]) AND ("Microscopy"[tiab] OR "Polymerase Chain Reaction"[tiab] OR "Immunologic Tests"[tiab] OR "Stool Examination"[tiab] OR "Serologic Tests"[tiab]))

- Region/Population (Location)

1. Malaysia [MeSH]
2. Southeast Asia [MeSH]
3. Asia [MeSH]
4. Developing Countries [MeSH]
5. Tropical Medicine [MeSH]
6. Malaysia [tiab]
7. Southeast Asia [tiab]
8. Asia [tiab]
9. Developing Countries [tiab]
10. Tropical Medicine [tiab]

**Preferred search term:** (("Malaysia"[MeSH] OR "Southeast Asia"[MeSH] OR "Asia"[MeSH] OR "Developing Countries"[MeSH] OR "Tropical Medicine"[MeSH]) AND ("Malaysia"[tiab] OR "Southeast Asia"[tiab] OR "Asia"[tiab] OR "Developing Countries"[tiab] OR "Tropical Medicine"[tiab]))

- Combined proposed search term will be used:

1. (Intestinal Protozoan Infections OR/AND Giardiasis OR Cryptosporidiosis OR Amebiasis) AND (Epidemiology OR Prevalence) AND (Detection Methods OR Digestive System OR Microscopy OR Immunologic Tests OR Polymerase Chain Reaction) AND (enteric OR diarrh* OR intestinal OR protozoa) AND (infection OR protozoa* OR parasit*) AND (in Malaysia OR Southeast Asia OR Asia)”.
2. The exploded versions of Medical Subject Heading or MeSH will be used for first theme. All themes will be searched for using “AND” in combination. The search, the evaluation of the titles and abstracts, and the review of the complete texts will be carried out by an independent author. After removing duplicates and irrelevant entries, the reference lists of the articles received were checked for further studies that were not found in the database search.
