# Supplementary material for: Epidemiological Characteristics of Intestinal Protozoal Infections and Their Risk Factors in Malaysia: Systematic Review and Meta-Analysis Protocol
Source: JMIR Res Protoc. 2025 Apr 4;14:e66350. doi: 10.2196/66350 (PMC12008696; doi:10.2196/66350)
Supplement: Multimedia Appendix 3 [file resprot_v14i1e66350_app3.docx]

**APPENDIX 3**

*Joanna Briggs Institute’s critical appraisal checklist [1] for studies of prevalence data*

| **No** | **Criteria** | **Response** |
| --- | --- | --- |
|  | Was the sample frame appropriate to address the target population? | *Yes, No, Unclear, Not applicable* |
| 2. | Were the study participants sampled in an  appropriate way? | *Yes, No, Unclear, Not applicable* |
| 3. | Was the sample size adequate? | *Yes, No, Unclear, Not applicable* |
| 4. | Were the study subjects and the setting described in detail? | *Yes, No, Unclear, Not applicable* |
| 5. | Was the data analysis conducted with sufficient coverage of the identified sample? | *Yes, No, Unclear, Not applicable* |
| 6. | Were valid methods used for the identification of the condition? | *Yes, No, Unclear, Not applicable* |
| 7. | Was the condition measured in a standard, reliable way for all participants? | *Yes, No, Unclear, Not applicable* |
| 8. | Was there appropriate statistical analysis? | *Yes, No, Unclear, Not applicable* |
| 9. | Was the response rate adequate, and if not, was the low response rate managed appropriately? | *Yes, No, Unclear, Not applicable* |

**REFERENCES**

1. Munn Z, Moola S, Lisy K, Riitano D, Tufanaru C. Methodological guidance for systematic reviews of observational epidemiological studies reporting prevalence and cumulative incidence data. Int J Evid Based Healthc. 2015;13(3):147-53.
