# Supplementary material for: Epidemiological Characteristics of Intestinal Protozoal Infections and Their Risk Factors in Malaysia: Systematic Review and Meta-Analysis Protocol
Source: JMIR Res Protoc. 2025 Apr 4;14:e66350. doi: 10.2196/66350 (PMC12008696; doi:10.2196/66350)
Supplement: Multimedia Appendix 4 [file resprot_v14i1e66350_app4.docx]

**APPENDIX 4**

*Newcastle - Ottawa Quality Assessment Scale (Cohort Studies)* [1,2].

Note: A study can be awarded a maximum of one star for each numbered item within the Selection and Outcome categories. A maximum of two stars can be given for Comparability

**Selection**

1. **Representativeness of the exposed cohort**
2. Truly representative of the average intestinal protozoan infection in the population (e.g., varied regions and socioeconomic backgrounds considered).**🟑**
3. Somewhat representative of the average intestinal protozoan infection in the population.**🟑**
4. Selected group of users (e.g., studies on specific settings like hospitals or clinics).
5. No description of the derivation of the cohort.
6. **Selection of the non-exposed cohort**
   1. Drawn from the same community as the exposed cohort.**🟑**
   2. Drawn from a different source (e.g., a geographically or demographically distinct population).
   3. No description of the derivation of the non-exposed cohort.
7. **Ascertainment of exposure**
   1. Secure record (e.g., validated diagnostic methods such as molecular techniques).**🟑**
   2. Structured interview (e.g., caregiver-reported or self-reported infections).**🟑**
   3. Written self-report.
   4. No description.
8. **Demonstration that outcome of interest was not present at start of study**
   1. Yes (e.g., initial screening excludes cases with prior intestinal protozoan infections).**🟑**
   2. No.

**Comparability**

1. **Comparability of cohorts on the basis of the design or analysis**
   1. Study controls for key confounders (e.g., socioeconomic status, diagnostic methods).**🟑**
   2. Study controls for additional factors (e.g., regional variation, immunocompromised status).**🟑**

**Outcome**

1. **Assessment of outcome**
   1. Independent blind assessment (e.g., diagnostic validation by independent researchers).**🟑**
   2. Record linkage (e.g., medical records for infections).**🟑**
   3. Self-report.
   4. No description.
2. **Was follow-up long enough for outcomes to occur**
   1. Yes (adequate follow-up period for detecting intestinal protozoan infection outcomes).**🟑**
   2. No.
3. **Adequacy of follow-up of cohorts**
   1. Complete follow-up – all subjects accounted for.**🟑**
   2. Small number lost to follow-up, unlikely to introduce bias (>90% follow-up or clear description of losses).**🟑**
   3. Follow-up rate <90% or no description of losses.
   4. No statement.

**REFERENCES**

1. Wells, G., Shea, B., O’Connell, D., Peterson, J., 2000. The Newcastle-Ottawa Scale (NOS) for assessing the quality of nonrandomised studies in meta-analyses [WWW Document]. Ottawa, Ottawa Hosp. Res. Inst.
2. Wells, G.A., et al., 2012. The Newcastle-Ottawa Scale (NOS) for assessing the quality if nonrandomized studies in meta-analyses. Evid. based public Heal. https://doi.org/10.2307/632432
